# Can People With Higher Versus Lower Scores on Impression Management or Self-Monitoring Be Identified Through Different Traces Under Faking?

**DOI:** 10.1177/00131644231182598

**Published:** 2023-07-02

**Authors:** Jessica Röhner, Philipp Thoss, Liad Uziel

**Affiliations:** 1 University of Bamberg, Germany; 2 Bar-Ilan University, Israel

**Keywords:** faking, response patterns, machine learning, impression management, self-monitoring

## Abstract

According to faking models, personality variables and faking are related. Most prominently, people’s tendency to try to make an appropriate impression (impression management; IM) and their tendency to adjust the impression they make (self-monitoring; SM) have been suggested to be associated with faking. Nevertheless, empirical findings connecting these personality variables to faking have been contradictory, partly because different studies have given individuals different tests to fake and different faking directions (to fake low vs. high scores). Importantly, whereas past research has focused on faking by examining test scores, recent advances have suggested that the faking process could be better understood by analyzing individuals’ responses at the item level (response pattern). Using machine learning (elastic net and random forest regression), we reanalyzed a data set (*N* = 260) to investigate whether individuals’ faked response patterns on extraversion (features; i.e., input variables) could reveal their IM and SM scores. We found that individuals had similar response patterns when they faked, irrespective of their IM scores (excluding the faking of high scores when random forest regression was used). Elastic net and random forest regression converged in revealing that individuals higher on SM differed from individuals lower on SM in how they faked. Thus, response patterns were able to reveal individuals’ SM, but not IM. Feature importance analyses showed that whereas some items were faked differently by individuals with higher versus lower SM scores, others were faked similarly. Our results imply that analyses of response patterns offer valuable new insights into the faking process.

Imagine you work for the police as a forensic psychologist and that you know that traces from a crime scene provide information about the personality traits of the perpetrators of the crime. You only have the traces of the perpetrators from the crime scenes to predict the perpetrators’ profiles, and you are now supposed to describe their personalities as precisely as possible. Thus, you try to predict the personalities of the perpetrators from their traces. To accomplish this task, you evaluate several crime scenes with different perpetrators. If your profiles fit and the perpetrators are justifiably convicted (on the basis of objective evidence), you know that the traces at the crime scenes are indeed associated with the personality traits. In the current study, we took this approach and applied it to the prediction of personality traits from the traces that are left by fakers.

## The Facts of the Case: Faking

Faking is part of the broader phenomenon of response distortion in psychological assessment (e.g., Röhner & Schütz, 2020).

Faking represents a response set aimed at providing a portrayal of the self that helps a person to achieve personal goals. [It] occurs when this response set is activated by situational demands and person characteristics to produce systematic differences in test scores that are not due to the attribute of interest. (Ziegler et al., 2012, p. 8)

Because faking often results in changes in test scores and rank orders, it is a serious problem that can impair the validity of tests (e.g., Salgado, 2016; see Ziegler et al., 2012, for an overview). Thus, understanding faking is an important challenge for psychological assessment (see Table 1 for an overview of the key messages).

**Table 1.** Summary Table with Key Messages.

Research topic	Key message
Defining faking	• Part of the broader phenomenon of response distortion^a^ • Response set aimed at providing a self-portrayal that helps a person achieve personal goals • Causes artificial changes in test scores and rank orders and impairs the validity of tests
Traces of faking and faking detection	• High scores on scales that aim to measure the tendency to create favorable impressions • Difference scores on total test scores • Traces at the item level as revealed by IRT models and machine learning
Individual differences in faking	• Personality traits impact faking, such that some individuals fake differently than others • Individuals with higher scores on IM are typically described as fakers, but recent findings suggest that this might not be the case • SM is also typically associated with faking, but the findings have been inconsistent • A neglected area of exploration in the study of associations between traits and faking is that traits might reveal differences only in faking patterns, not in total scores
Relevance of personality with respect to faking detection	• Personality may play a role in faking detection if different individuals fake *differently* (i.e., leave different traces) • Associations between personality and faking may appear only on selected items and not on test scores • Consequently, fakers with higher scores on IM or SM might be detected by different items than those with lower scores on IM or SM

*Note*. IRT = item response theory; IM = impression management; SM = self-monitoring.

a References are presented in the main text.

## Traces of Faking and Faking Detection

When trying to find the differences between faked and nonfaked responses, it is important to investigate the *traces* that fakers leave. Traces can be understood as indicators of faking. As such, they stimulate the development of approaches for detecting faking (i.e., identifying faking on the basis of the traces fakers leave). The goal of detecting faking reliably has been pursued for more than 100 years (Sackett et al., 2017). Accordingly, a variety of approaches have been tested. Although a detailed and complete list of them is beyond the scope of this article, in the following, we will nevertheless provide an overview of the most prominent traces that have been used in faking research along with prominent approaches in faking detection. So far, none of these procedures have been widely accepted (Röhner et al., 2022). Also, none of them work without misclassification, which indicates that there are still factors that are not understood in the detection of faking.

### Scales to Measure the Tendency to Create Favorable Impressions

The most prominent, traditional method for detecting traces of faking involves scores on *scales that aim to measure the tendency to create a favorable impression* (e.g., Paulhus, 2017). Designed to measure tendencies to deceive others in a socially desirable manner, impression management (IM) scales often go by other names, such as lie scales, other-deception scales, validity scales, or social desirability scales (e.g., Eysenck et al., 1985; Sackeim & Gur, 1978; Stöber, 2001). IM scales usually ask about favorable but implausible behaviors (e.g., “When I hear people talking privately, I avoid listening”) or about relatively frequent unfavorable behaviors (e.g., “I never swear”). A high score on an IM scale results from endorsing the favorable items and denying the unfavorable items and has been considered to offer a trace of faking.

The application of IM scales to detect biased responses is prevalent in both research and practical contexts, either as stand-alone measures or as part of a broader inventory (see Goffin & Boyd, 2009; Uziel, 2010, for reviews). However, developments in research have raised doubts about the utility of IM scales as measures of response biases (e.g., Connelly & Chang, 2016; de Vries et al., 2014; Lanz et al., 2022; Uziel, 2010). Research has demonstrated that such scales often fail as measures of faking and that, instead, they measure (partially or mostly) personality substance, such as conscientiousness, agreeableness, honesty-humility, and self-control (e.g., de Vries et al., 2014; Uziel, 2010). Thus, this approach for detecting faking has been criticized for carrying the risk of erroneously suspecting people who score high on certain personality traits (e.g., conscientiousness) to be fakers (Uziel, 2010, see also Connelly & Chang, 2016; Lanz et al., 2022).^1^

### Difference Scores

An alternative traditional approach involves the use of *difference scores* (e.g., Ferrando & Anguiano-Carrasco, 2011; Röhner & Schütz, 2020). This approach is usually implemented to study faking experimentally and usually focuses on differences in test scores between faking and nonfaking conditions (e.g., Alliger & Dwight, 2000; McDaniel et al., 2009; Röhner et al., 2011; Viswesvaran & Ones, 1999; Wood et al., 2022). Although difference scores have been criticized in the past (e.g., Bereiter, 1963), recent research has demonstrated that, under certain conditions, they are a reasonably reliable measure^2^ (e.g., Gollwitzer et al., 2014; Trafimow, 2015; Trafimow, 2019).

#### Scale Level Versus Item Level

Difference scores were usually used at the *test score* level. However, faking models (e.g., Goffin & Boyd, 2009) and data (Brown & Böckenholt, 2022) have suggested that there is no constant amount of faking that is added to (or subtracted from) all items but that the amount of faking differs from item to item. This phenomenon is called intermittent faking (Brown & Böckenholt, 2022). In this line of thinking, recent studies have demonstrated that test scores are not able to capture the complete picture of faking (e.g., Calanna et al., 2020; Röhner et al., 2022). Relatedly, studies have shown that looking at individuals’ responses to each individual item on the to-be-faked measure (in its entirety called individuals’*response patterns*; e.g., Calanna et al., 2020) may offer better insights into faking because individual items include information about traces of faking that are washed out in test scores (e.g., Böckenholt, 2013, 2017; Calanna et al., 2020; Lee et al., 2022; Röhner et al., 2022; Sun et al., 2022).

In this line of research, by using item response theory (IRT) models, studies have demonstrated that faking detection is improved by dissecting individual item responses into item response trees and using the item response trees in modeling (e.g., Böckenholt, 2013, 2017; Lee et al., 2022; Sun et al., 2022). Relatedly, research using machine learning and item responses has successfully detected faking with high levels of accuracy (e.g., Calanna et al., 2020; Röhner et al., 2022). Taken together, the results point to the necessity of investigating responses on individual items as the more reliable approach for tracing faking.

In the current study, we sought to integrate previous research that focused on difference scores (e.g., Alliger & Dwight, 2000; McDaniel et al., 2009; Röhner et al., 2011; Viswesvaran & Ones, 1999; Wood et al., 2022) with current approaches that take an item-level approach to trace faking (e.g., Böckenholt, 2013, 2017; Brown & Böckenholt, 2022; Lee et al., 2022; Sun et al., 2022).

## Do all Individuals Leave the Same Traces When Faking?

The definition by Ziegler et al. (2012) indicates that, besides situational demands (e.g., high-demand situations, such as personnel selection processes), there are also characteristics of the person that may shape their faking. In other words, it is possible that not all individuals fake in a similar way (e.g., due to their personality traits). From the stance of faking detection, different individuals can be expected to leave different traces of faking, and thus, different individuals who fake cannot be detected in the same way.

Indeed, personality traits have a long tradition of being studied in relation to faking (e.g., Goffin & Boyd, 2009; Levashina & Campion, 2006; McFarland & Ryan, 2000; Roulin et al., 2016). Faking has been found to be related to several personality dispositions. Central among them are IM, which taps a habitual tendency to present oneself in a favorably biased manner (Paulhus, 1984), and self-monitoring (SM), which reflects a tendency to strategically adapt one’s behavior to situational demands (Snyder, 1974).

### Faking and IM

IM has traditionally been defined as a habitual (i.e., trait-like) tendency to distort one’s self-presentation to convey a favorable impression (e.g., Paulhus, 2017). Compared with people who score lower on IM, people scoring higher on IM present a favorably biased image of themselves consciously and deliberately, with an emphasis on appearing, for example, moral, communal, and dutiful (Paulhus & John, 1998).

However, broad quantitative and narrative reviews have arrived at the same conclusion over the years, building on diverse sources of information that have converged in showing that IM scores are not reliable moderators or suppressors of self-reports but that they measure substantive content (Connelly & Chang, 2016; de Vries et al., 2014; Uziel, 2010, 2014). Moreover, direct tests of faking have also indicated that individuals’ scores on IM scales are not related to faking (e.g., Mersman & Shultz, 1998; Pauls & Crost, 2005; Zettler et al., 2015).

IM scales were developed to measure bias, and IM should be related to faking (although recent research has called this association into question). Notwithstanding, previous research has focused on investigating the association between IM and faked *test scores* but not between IM and faked *response patterns*. As mentioned above, research has recently demonstrated that faking takes place *on some but not all items* (Brown & Böckenholt, 2022) and that test scores are not able to capture the complete picture of faking (e.g., Calanna et al., 2020; Röhner et al., 2022). Thus, it may be possible that there is an association that is covered up when faking on test scores is investigated but is revealed at the level of response patterns. It may well be the case that, for example, individuals with higher scores on IM compared with individuals with lower scores on IM do not simply fake more strongly on all items but only on specific items. This difference would not automatically translate into differences that can be detected in test scores (e.g., if both groups fake to the same extent but on different items). In sum, individuals with higher scores on IM may exhibit greater faking on only selected items instead of faking in a blatant way across all items. Thus, response patterns of people with higher versus lower scores on IM should be tested to investigate whether these response patterns differ during faking.

### Faking and SM

SM is the ability to manage and control one’s expressive behaviors or self-presentation and evaluate others’ reactions to achieve a desired impression (Snyder, 1974; see also Fuglestad & Levert, 2022). Whereas people with lower SM scores tend to project a stable self-image in diverse situations, people with higher SM scores adapt their appearance and actions to fit the respective situation (Day & Schleicher, 2006; Fuglestad & Snyder, 2009). Consequently, people with higher (but not lower) scores on SM endorse items such as, “In different situations and with different people, I often act like very different people,” or “I’m not always the person I appear to be.” That is, by definition, SM often serves to mask a person’s true self and convey inaccurate social signals (Snyder, 1974).

On the basis of this line of thinking, it can be reasoned that people who score higher (but not lower) on SM will be better able to adapt to situations involving faking and will exhibit faking to a greater extent when required to do so. Also of interest is the association of SM with authenticity. According to Snyder (1974), individuals who score lower on SM are more authentic in their self-presentation, whereas individuals who score higher on SM have a more volatile self-perception. Little research has addressed this association directly, generally supporting this assertion (e.g., Pillow et al., 2017; but see Laux & Renner, 2002, for mixed results). Notwithstanding, current associations with authenticity should be considered cautiously because they are based on self-reports and thus prone to bias.

Although several studies have suggested that SM is related to faking (e.g., Goffin & Boyd, 2009; McFarland & Ryan, 2000; Mueller-Hanson et al., 2006; Roulin et al., 2016), the empirical evidence has been inconsistent. Alongside findings associating SM with faking behavior (Schroeder & Cavanaugh, 2018), some studies have not found such a relationship (McFarland & Ryan, 2000; Mersman & Shultz, 1998; Mueller-Hanson et al., 2006).

The contradictory findings from these studies may be partly explained by differences in the study designs. For example, faking direction has been demonstrated to impact faking in several studies (e.g., Bensch et al., 2019; Röhner et al., 2022), and thus, not systematically controlling for faking direction (i.e., faking low vs. faking high scores) might lead to inconsistent findings. To obtain a more coherent picture of faking, especially in relation to the personality traits mentioned above, there is a need to explore faking using similar settings (Röhner et al., 2022). In addition, as for IM, studies have focused on investigating the relationships of SM to changes in *test scores* on the to-be-faked measure (e.g., Mersman & Shultz, 1998), but existing effects may be washed out if the test score is chosen as the measure for determining whether individuals with higher (vs. those with lower) SM scores fake to a greater extent because faking does not necessarily occur *on all items* (see Brown & Böckenholt, 2022).

Which items individuals view as relevant for faking could in turn be impacted by several factors, such as the described personality variables (e.g., for SM: the ability to manage and control one’s behavior and self-presentation). Thus, it is likely that individuals with higher scores on SM will exhibit greater faking on only selected items to give the desired impression instead of faking in a blatant way across all items. Thus, here too, analyzing individuals’ response patterns at the item level might offer valuable insights.

## Personality’s Relevance for Faking Detection

Despite the progress that past research has made in detecting faking using machine learning (e.g., Calanna et al., 2020; Röhner et al., 2022) or item response trees (e.g., Böckenholt, 2013, 2017; Lee et al., 2022; Sun et al., 2022), there are still no valid faking detectors that can identify fakers without restrictions. Thus, more insight into individual differences in faked responses seems relevant. Following this line of thinking, taking certain personality variables into consideration may provide valuable insights because traits may predispose individuals to specific faking patterns (i.e., faking on some of the items), which may help with faking detection. So far, this has not been done. As described above, IM and SM have a long tradition of being suggested to shape faking behavior. Thus, personality may play a role in faking detection because, if individuals fake *differently* according to their personality, they will leave different traces in their faked responses.^3^ If individuals leave different traces of faking on different items on the basis of their IM or SM, it is relevant for faking detection. In this case, fakers with higher scores on IM or SM might be revealed by different items than those with lower scores on IM or SM. If this is true, the findings would imply that personality should be taken into account when developing approaches to detect faking to improve faking detection.

On the basis of these theoretical, empirical, and methodological considerations, the present study was designed to investigate whether individuals with higher (vs. lower) scores on IM or SM can be characterized by different patterns of responses when they fake (i.e., whether they leave different traces of faking). We addressed this question by employing machine learning, which presents unique advantages for studying complex response patterns (Calanna et al., 2020).

## Analyzing Faked Response Patterns With Supervised Machine Learning

Machine learning has sparked immense interest recently and has been applied to several complex psychological problems (e.g., Calanna et al., 2020). Consequently, machine learning has also been successfully applied to the complex phenomenon of faking (Calanna et al., 2020; Röhner et al., 2022).

Research has shown that supervised machine learning is an effective way to investigate the complexity of individuals’ response patterns^4^ (Calanna et al., 2020; Röhner et al., 2022). With respect to faking research, faking can be modeled by the differences in responses given by individuals at baseline (i.e., nonfaking control condition) and in faking conditions (e.g., Röhner et al., 2013).^5^ Although difference scores can suffer from unreliability (Bereiter, 1963), they have been used frequently in faking research (Alliger & Dwight, 2000; Viswesvaran & Ones, 1999; Wood et al., 2022) because the unique conditions of the faking situation make it less likely for them to be unreliable. We explain this point in detail in the Method section.

Machine learning can be used to make predictions in classification and regression tasks (e.g., Calanna et al., 2020; Owens et al., 2022). When applying machine learning to regression tasks, the goal is to predict a continuous variable from several input variables (i.e., features; e.g., Speer et al., 2022). We wanted to investigate whether people with higher (vs. lower) scores on a given trait (e.g., SM) change their responses from baseline to faking in different ways and thus show different response patterns. If people differ with respect to how they fake because of their standing on a personality trait (e.g., their SM score), then machine learning will be able to predict that trait above chance levels.^6^ If machine learning is not able to do so, then the response patterns are comparable, and the personality trait in question does not play a significant role in faking. The advantage of this approach is that instead of looking at the mean faking levels of individuals varying in SM or IM (an artificial approach in instructed faking settings that are used most frequently in faking research),^7^ we specifically examined the items where the most faking occurred (i.e., data-driven) and tested whether we could differentiate between higher and lower scorers on SM or IM from individuals’ responses on these items. Thereby, we gained insight into the personality correlates where actual faking occurs.

Whether or not machine learning is able to predict the respective trait above chance levels can be evaluated by a bundle of performance measures (e.g., in our case, *R*^2^, the root mean square error [*RMSE*], the mean average error [*MAE*], and the mean square error [*MSE*]; Ayitey Junior et al., 2023; Iskandaryan et al., 2020) that are explained in detail in the Method section. Moreover, feature importance analyses allow researchers to obtain in-depth insights into which items were most important for the prediction (e.g., Vijayakumar & Cheung, 2018), that is, which items were faked differently by individuals with respect to their scores on the respective personality variable (e.g., the most important feature for predicting the SM of fakers is Item 3). Also, they can be used to make inferences about another relevant question: How do the responses of individuals with higher versus lower scores on the respective variable differ (e.g., individuals with higher scores on SM exhibit stronger faking on Item 3 than those with lower scores on SM).

In summary, an investigation of differences in response patterns (i.e., at the item level) might offer a more detailed picture of how personality variables contribute to faking than what can be inferred from analyses of differences in test scores (e.g., Brown & Böckenholt, 2022; see also Böckenholt, 2013, 2017; Calanna et al., 2020; Lee et al., 2022; Röhner et al., 2022; Sun et al., 2022). Moreover, investigating response patterns in the context of both personality traits (i.e., IM and SM) in the same setting on a single to-be-faked measure and for both faking directions (i.e., faking low and faking high; e.g., Röhner et al., 2022) sets the ground for a more accurate assessment of the relative roles of these personality variables in faking.

## The Present Study

This study was developed to systematically investigate whether the response patterns of individuals with higher versus lower scores on IM or SM differ from each other under faking conditions. We adopted this new item-level-based approach to achieve more fine-grained insights into the associations between faking behavior and personality traits (e.g., Brown & Böckenholt, 2022; see also Böckenholt, 2013, 2017; Calanna et al., 2020; Lee et al., 2022; Röhner et al., 2022; Sun et al., 2022). We asked individuals to fake on an extraversion scale because successful faking on extraversion has frequently been demonstrated, and both faking directions are plausible (e.g., McDaniel et al., 2009; Röhner et al., 2013; Röhner & Holden, 2022). Individuals were asked to fake either low or high scores and were not given a strategy or any information about how to do so. This so-called naive faking (see, e.g., Röhner et al., 2013; Röhner & Ewers, 2016a, 2016b) was expected to challenge individuals’ abilities so that the importance of the personality variables could be demonstrated as distinctively as possible. The research questions and hypotheses are as follows:

1. We aimed to investigate whether the response patterns of individuals with higher versus lower scores on IM would differ from each other when faking on extraversion. IM scales were specifically developed to measure bias, their sole purpose in science. On this basis, there is an expectation that they will be significantly related to faking. However, recent developments have called this association into question (e.g., Connelly & Chang, 2016; Zettler et al., 2015), but this research has focused on test scores. Because faking is an item-based process, we tested these contrasting hypotheses against each other while using new tools (e.g., by focusing on test items). If fakers leave different traces (i.e., different response patterns when faking on extraversion) on the basis of their IM scores, it should be possible to use the traces to reveal their IM scores above chance levels. We did not have an a priori prediction about which items would be most indicative of differences in faking, and thus, on the basis of previous research that has examined the concept of intermittent faking (Brown & Böckenholt, 2022), we conducted exploratory tests of differences on the items.
2. Because of the inconsistencies in previous findings, we wanted to investigate whether the response patterns of individuals with higher versus lower scores on SM would differ from each other when faking on extraversion. Similar to IM, the literature on SM implies that individuals with higher SM scores (vs. individuals with lower SM scores) should be more likely to fake (e.g., Goffin & Boyd, 2009; McFarland & Ryan, 2000; Mueller-Hanson et al., 2006; Roulin et al., 2016). Here too, findings were not entirely consistent and were based on test scores, although faking is better revealed by item response patterns than by total test scores (e.g., Brown & Böckenholt, 2022; see also Böckenholt, 2013, 2017; Calanna et al., 2020; Lee et al., 2022; Röhner et al., 2022; Sun et al., 2022). We expected that individuals with higher versus lower SM scores would leave different traces (i.e., response patterns when faking on extraversion) and therefore that we would be able to use their traces to predict their SM scores above chance levels with machine learning. We did not have an a priori prediction about which specific items would be most indicative of differences in faking, and thus, on the basis of previous research that has examined the concept of intermittent faking (Brown & Böckenholt, 2022), we conducted exploratory tests of differences on the items.

## Method

### Data Set

To test our predictions, we reanalyzed a data set (*N* = 300) that was previously collected under the supervision of the lead author in an investigation of faking on measures of extraversion (Allramseder, 2018; Dirk, 2017; Doukas, 2017; Hütten, 2018; Möller, 2017).^8^ The analyses reported in the present report are original and were not reported in previous studies. Individuals with missing data were excluded from the analyses. Ten individuals were excluded because they did not participate at the second occasion at all. Thirty individuals were excluded because they had at least one missing value in one item. Thus, about 13% were excluded from further analyses. We decided to adopt this conservative requirement because we wanted to assess faking as purely as possible without mixing it up with careless responding (Schroeders et al., 2022). Thus, the final data set comprised 260 individuals (257 students; 191 women, 69 men, 3 diverse/no response; average age: 21.22 years, *SD* = 4.74).

We chose this data set for several reasons: First, unlike most studies on faking that focus on one direction of faking (usually the faking of high scores), this data set included both the faking of high scores and the faking of low scores. Individuals were randomly assigned to one of these groups after a baseline assessment. Because our interest was in the impacts of faking high and faking low, it was necessary for both faking directions to be included in the same data set. Second, extraversion is a construct that has frequently been investigated in previous faking research, and both faking directions (i.e., high and low) are plausible for this construct (e.g., McDaniel et al., 2009; Röhner et al., 2013; Röhner & Thoss, 2018). Last but not least, because we had 260 individuals in the study, even after excluding individuals in the control condition, there were more than 88 individuals (faking low condition) or 86 individuals (faking high condition) in each regression model. Such numbers can be considered sufficient for machine learning, although research has also demonstrated that machine learning can be successful with smaller samples (see, e.g., Li et al., 2017). We conducted a power analysis for a robust analysis of covariance (ANCOVA), which was computed to assess the manipulation check analyses, using the ancmg1.power function by Wilcox (2022).^9^ The power analysis revealed a power > .98 for the robust ANCOVA to detect a moderate effect size at an alpha level of .05 (*N* = 260).

### Procedure

Individuals took part in the study in exchange for personal feedback or partial university course credit. They completed the extraversion scale (Borkenau & Ostendorf, 2008) twice. On the first occasion (i.e., baseline assessment), they completed the extraversion scale under standard instructions. Then, in a random order, they completed the IM scale and the SM scale. On the second occasion, 2 days later, individuals were randomly assigned to one of three conditions (i.e., control, the faking of high scores, or the faking of low scores). Individuals in the control condition (*n* = 86) were again given standard instructions, whereas fakers were asked to fake either high scores (*n* = 86) or low scores (*n* = 88) on the extraversion scale according to a personnel selection scenario. So that individuals’ faking could be assessed as it would normally occur in a personnel context, fakers were not provided with any strategies on how to fake (i.e., *naive faking*; see, e.g., Röhner et al., 2013, 2023 for further information). In the instructions for the faking of high scores, individuals were asked to imagine that they had been unemployed for 1 year and had now received a very attractive job offer. They were asked to fake high on extraversion to maximize their chances of being offered the job. The instructions for the faking of low scores included the description of a very unattractive job offer. To avoid being offered the job, individuals were asked to fake low extraversion.^10^

### Ethics Approval

The original study for which the current data were collected was reviewed by the Ethics Committee of the Technical University of Chemnitz, Germany, and approval was granted (approval number: V-151-BM-JR-IAT-26072016). The study was conducted in accordance with the ethical standards of the 1964 Declaration of Helsinki and its later amendments or comparable ethical standards.

### Consent to Participate

Written informed consent was provided by the individuals.

### Measures

**Figure 1.**
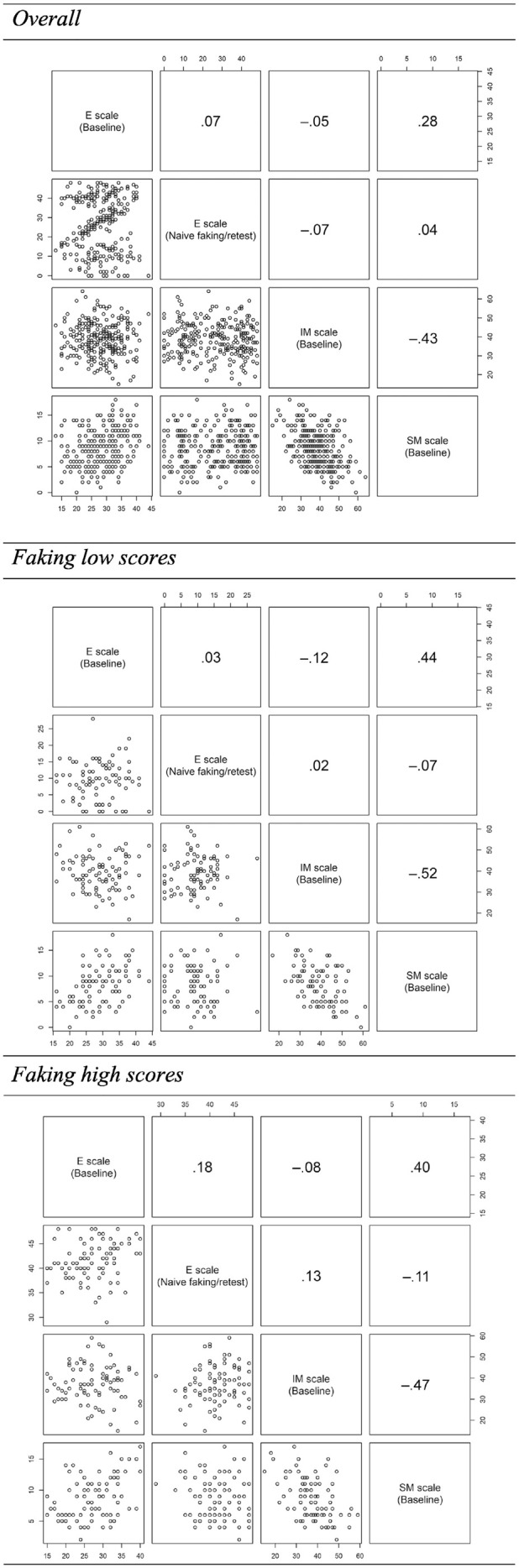
Sample-Based as Well as Group-Based Correlations and Scatterplots Between the E Scale, IM Scale, and SM Scale. *Note.* E = extraversion; IM = impression management; SM = self-monitoring. *p* ≤ .05 for *r* ≥ |.28|.

Individuals worked on the scales described below. An overview of the descriptive statistics of these scales is presented in Table 2. For correlations and scatterplots between the scales, see Figure 1.

**Table 2.** Descriptive Statistics for the E Scale, IM Scale, and SM Scale.

		Experimental group		
	Measurement occasion	Faking low	Control group	Faking high
Scale		*M*_sum_ *(SD)*	*M*_sum_ *(SD)*	*M*_sum_ *(SD)*
E	Baseline	28.98 (6.31)	27.77 (6.34)	27.48 (6.03)
E	Naive faking/retest	9.27 (5.62)	27.85 (6.36)	41.44 (3.89)
IM	Baseline	39.24 (8.50)	39.55 (8.81)	37.27 (8.84)
SM	Baseline	8.24 (3.73)	8.88 (3.50)	8.66 (3.36)

*Note*. E = extraversion; IM = impression management; SM = self-monitoring. *N* = 260 (*n* faking low = 88, *n* control group = 86, and *n* faking high = 86).

#### Extraversion Scale (E Scale)

Individuals worked on the respective scale from the NEO-Five Factor Inventory (Borkenau & Ostendorf, 2008; English version: Costa & McCrae, 1992). This scale consists of 12 items that are answered on a 5-point rating scale ranging from 0 (*strongly disagree*) to 4 (*strongly agree*). Example items are, “I really enjoy talking to people” and “I am a very active person.” Scale characteristics and Cronbach’s alpha reliability scores for the baseline assessment (*M*_sum_^11^ = 28.08, *SD*_sum_ = 6.24, α = .79) were comparable to Borkenau and Ostendorf’s (2008) values (*M*_sum_ = 28.38, *SD*_sum_ = 6.70, α = .80). Faking led to typical consequences (e.g., Salgado, 2016): The means decreased, and the standard deviations and reliability scores increased (*M*_sum_ = 26.03, *SD*_sum_ = 14.38, α = .96). *M* and *SD* based on average scores were *M*_a_ = 2.34 (*SD*_a_ = 1.06) for the baseline assessment and *M*_a_ = 2.17 (*SD*_a_ = 1.46) for faking in our study.

#### IM Scale

The respective subscale from the German adaption of the Balanced Inventory of Desirable Responding (Musch et al., 2002; English version: Paulhus, 1994) was used to assess IM. The subscale consists of 10 items rated on a 7-point scale ranging from 1 (*strongly disagree*) to 7 (*strongly agree*). Example items are, “I sometimes tell lies if I have to” and “I have taken sick-leave from work or school even though I wasn’t really sick.” The scale characteristics in the present study were *M*_sum_ = 38.69, *SD*_sum_ = 8.74, and α = .63. Therefore, they were comparable to Musch et al.’s (2002) values of *M*_sum_ = 31.40, *SD*_sum_ = 9.20, and α = .65. *M* and *SD* based on average scores were *M*_a_ = 3.87 (*SD*_a_ = 2.06) in our study.

#### SM Scale

Individuals completed the German adaption of the SM Scale (Graf, 2004; English version: Snyder, 1974; Snyder & Gangestad, 1986). It consists of 18 items that are rated as *true* or *false*. Example items are: “In different situations and with different individuals, I often act like very different people” and “I’m not always the person I appear to be.” The scale characteristics in the present study were *M*_sum_ = 8.59, *SD*_sum_ = 3.53, and α = .73, and were therefore comparable to the values from other studies that have used this scale (*M*_sum_ = 9.19, *SD*_sum_ = 3.26, α = .67; e.g., Winter, 2020). *M* and *SD* based on average scores were *M*_a_ = 0.48 (*SD*_a_ = 0.50) in our study.

### Analytical Approach

#### Manipulation Check

To check whether individuals in the faking groups were able to fake on the E scale and whether their faking scores still differed when the baseline scores were controlled for, we computed a robust ANCOVA (Wilcox, 2005) on the extraversion score. We used the faked score on the E scale as the dependent variable, the experimental group as the independent variable, and the score on the E scale at baseline as the covariate. As expected, the significant differences between the trimmed means in all design points revealed that individuals in the faking conditions were motivated and able to fake on the E scale (Table 3).

**Table 3.** Results of the Robust ANCOVA on the E Scale Scores.

				95% CI	
Points chosen	Group	*n*	Psi hat	LL	UL
	Faking low	54	−16.15	−18.92	−13.59
24	Control	65	−31.97	−34.69	−29.75
	Faking high	57	−15.82	−17.79	−13.97
	Faking low	41	−18.92	−21.22	−16.59
28	Control	55	−32.02	−34.31	−29.91
	Faking high	42	−13.10	−14.95	−11.38
	Faking low	52	−21.11	−23.57	−18.95
33	Control	61	−32.28	−35.03	−29.88
	Faking high	45	−11.17	−13.30	−8.90

*Note.* E = extraversion; CI = confidence interval; LL = lower limit; UL = upper limit.

#### Data Preparation: Computation of Difference Scores

Difference scores for the responses on the items on the E scale were calculated by subtracting the baseline score on the respective item from the faked score on this item for the faking of high scores (see, e.g., Röhner et al., 2013, 2023). The computation was vice versa for the faking of low scores; e.g., Röhner et al., 2023).^12^

Concerns have been voiced with respect to the reliability of difference scores (Bereiter, 1963). However, Trafimow (2015) and Gollwitzer et al. (2014) argued against a blanket recommendation not to use difference scores and showed that the reliability of difference scores depends on a complex interaction of factors (e.g., low reliability of measures are related to low reliability of difference scores, large differences in standard deviations between measurement occasions will increase the reliability of difference scores) and thus demonstrate that the bad reputation of difference scores is often unwarranted.

From a theoretical stance, difference scores in faking research are not likely to have low reliability. First, faking research has revealed strong treatment effects (e.g., McDaniel et al., 2009; Röhner et al., 2011), which cause large differences in standard deviations between measurement occasions, thus increasing reliability (see Gollwitzer et al., 2014). Therefore, difference scores in faking research (that demonstrate strong treatment effects) could be anticipated to be reliable, and their frequent and successful application in faking research attests to this (e.g., Alliger & Dwight, 2000; Röhner et al., 2011; Viswesvaran & Ones, 1999; Wood et al., 2022). Second, based on Trafimow’s (2015) results, several aspects of the specific research condition (i.e., faking) point to the fact that difference scores should not be unreliable here. For example, the reliabilities of the measures usually increase (e.g., Salgado, 2016) under faking (i.e., individual tests), and such an increase occurred in our study (baseline: *r* = .78 and faking *r* = .97). High reliabilities on the individual tests have a positive impact on the reliability of the difference scores (Trafimow, 2015). Thus, low reliabilities of individual tests should usually not be a topic in faking research, at least if the individual tests have acceptable reliabilities per se (at the baseline assessment).

From an empirical stance, Trafimow’s (2015) formulas allow the reliability of difference scores to be determined only at a test score level.^13^ To assess the situation with respect to our study, we therefore followed Trafimow’s (2019) argumentation that the algebraic rearrangement of the equation from classical test $\rho_{\mathrm{XY}}=\rho_{T_{X}T_{Y}}\sqrt{\rho_{XX^{\prime }}\rho_{YY^{\prime }}}$ , which is known as the disattenuation equation ρTXTY=ρXY/ρXX'ρYY' , implies that the smaller ρXX' (i.e., the reliability of the difference scores), the larger $\rho_{T_{X}T_{Y}}$ (i.e., the correlation of the true scores) would have to be to nevertheless obtain a reasonable value for $\rho_{\mathrm{XY}}$ . We applied the disattenuation equation to analyze the expected reliability of the difference scores at the item level. Therefore, we added the correlations between the difference scores at the item level and the IM scale (or SM scale), the reliability of the IM scale (or SM scale), and simulated values for the reliability of the difference scores (i.e., low = .40, moderate = .70, high = .80) to the disattenuation equation and retrieved the correlations of the true scores. The results showed that even if the reliability of the difference scores had been reduced, the true correlation coefficient would have been larger, which could also be expected from the classical test theory equation. The results can be found in Table S1 in the Supplement on the OSF (https://osf.io/ujvwd/).

#### Data Analyses With Supervised Machine Learning

To investigate the ability of machine learning to reveal whether fakers have higher or lower scores on our focal traits (i.e., IM or SM), we used elastic net regression (Zhou & Hastie, 2005) and random forest regression (Breiman, 2001).^14^ We decided to use two approaches to examine the results with regard to their convergence. We decided to apply these two popular approaches because they have been widely and successfully applied before and because they represent two types of regression (i.e., black box method [random forest] vs. regression-based method [elastic net]; see Vijayakumar & Cheung, 2018). In addition, both elastic net regression and random forest regression are capable of accommodating highly correlated features (Breiman, 2001; Zhou & Hastie, 2005), whereby elastic net regression, in particular, is highly effective at dealing with highly correlated features (Owens et al., 2022), as could be expected in our case (because the responses on the extraversion items all belong to the E scale).

Elastic net regression and random forest regression were applied to individuals’ response patterns (i.e., differences in the item scores between baseline and faking). In each case, all the items from the E scale were used because this procedure is superior to the use of test scores (see, e.g., Calanna et al., 2020; Röhner et al., 2022). Codes for analyses are stored on the OSF (https://osf.io/ujvwd/).

##### Multilayer Cross-Validation

**Figure 2.**
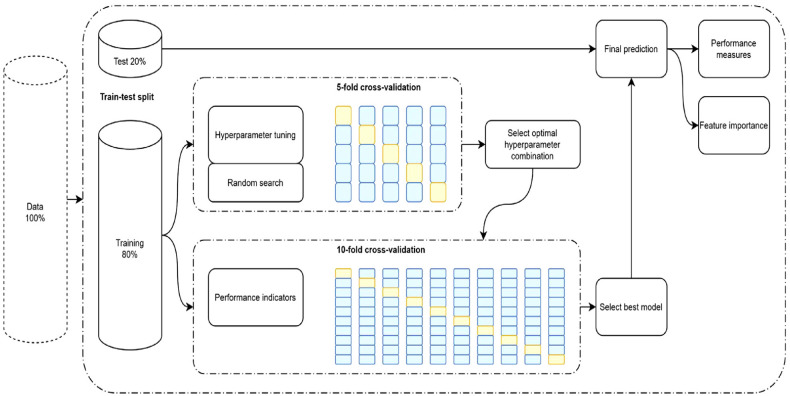
Cross-Validation Framework

To ensure the generalizability of the results, we followed previous approaches and recommendations (e.g., Calanna et al., 2020; Röhner et al., 2022; Zhou et al., 2015) and adopted a multilayer cross-validation procedure. We ran a five-fold cross-validation to tune the algorithms and additionally ran another 10-fold cross-validation to estimate their performance (Cawley & Talbot, 2010). The training data and test data were independent from each other in every fold (i.e., 80%/20% data split). This was true for the five-fold cross-validation that was used to tune the algorithms and also for the 10-fold cross-validation that was used to estimate performance. Figure 2 shows the cross-validation framework. We used a random search to tune the hyperparameters because a random search has been shown to be more effective than a traditional grid search (Bergstra & Bengio, 2012; Owens et al., 2022). The random search simply selects random combinations of hyperparameters and tests each of them. Hyperparameters are adjustable parameters that are tuned (i.e., modified) to obtain a model with optimal model performance (e.g., Speer et al., 2022). The hyperparameters for the elastic net regression were λ and α. Lambda is a complexity parameter that can be larger or equal to zero. It determines the degree to which regression weights should be penalized (e.g., Speer et al., 2022). Elastic net regression can be divided into two special regressions (i.e., ridge regression, Hoerl & Kennard, 1970; LASSO regression, Tibshirani, 1996). Thus, α represents a parameter that determines the degree to which the formula is a mix of ridge regression (i.e., α equals 0) and LASSO regression (i.e., α equals 1) and can therefore be thought of as a mixing parameter. The hyperparameters for the random forest regression were *mtry* and *ntree*. Mtry determines the number of predictors to compare at each split of a tree (e.g., Vijayakumar & Cheung, 2018). Ntree represents the number of decision trees that are created (e.g., Liaw & Wiener, 2002). The best set of hyperparameter combinations was selected by using the *RMSE* to maximize the absolute predictive fit (e.g., Kuhn & Wickham, 2020).

##### Performance Evaluation

The performance of regressions is typically evaluated with the following performance indices: *R*^2^, *RMSE, MAE*, and *MSE*, whereby *R*^2^ and *RMSE* are used most frequently (e.g., Ayitey Junior et al., 2023; Iskandaryan et al., 2020). *R*^2^ represents a measure of relative improvement in prediction over the mean model. *RMSE* is a measure of absolute predictive fit. *MAE* is the mean absolute error between predicted and actual outcomes. *MSE* is the mean square error between predicted and actual outcomes. The performance indices we used can be divided into two groups. The first group explains the strength of the relationship between predictive models and the target variables (i.e., *R*^2^, where a larger score with a maximum of one indicates a better fit of the model and negative scores indicate a clearly inappropriate fit of the model; see Iskandaryan et al., 2020; Kvålseth, 1985). The second group describes the difference between prediction results and true values (i.e., *RMSE*, *MAE*, and *MSE*, where smaller values indicate better fit; see Iskandaryan et al., 2020). All four indices can be used to assess the model’s performance on the same data set, but only *R*^2^ can be used to assess the model’s performance on different data sets (e.g., Chicco et al., 2021).

##### Feature Importance

**Figure 3.**
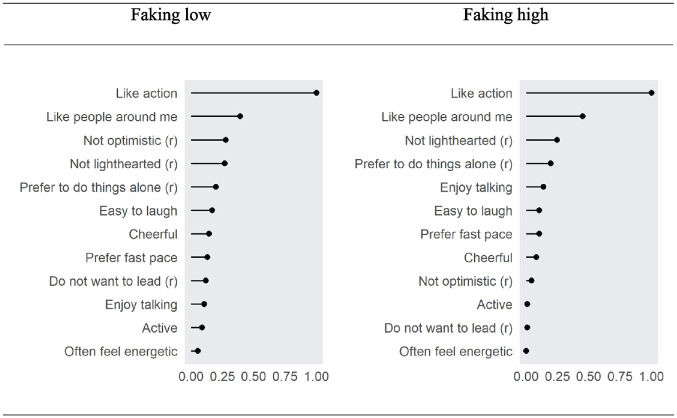
Feature Importance When Predicting Participants’ Scores on SM With Respect to Faking Low and High Scores on Extraversion *Note*. Feature importance is sorted in descending order in each case.

To gain insight into the black box of how people with higher scores on the respective focal trait fake differently from people with lower scores on it, we explored the features that were used by the regressions to predict the respective focal trait (see Figure 3). The features are the response patterns of individuals on the extraversion items (i.e., differences between faking and baseline).

##### Controlling for Faking Direction

Because research has demonstrated that faking direction (i.e., faking low vs. faking high) impacts faking (e.g., Bensch et al., 2019; Röhner et al., 2022), a finding that might at least partly explain the contradictory findings of past studies that did not systematically control for both faking directions, we used separate analyses to consider the impact of faking direction on faking.

### Statistical Packages

All analyses were computed with *R* (4.1.3) using the following packages: ggh4x (0.2.1), ggtext (0.1.1), haven (2.5.0), here (1.0.1), patchwork (1.1.1), psych (2.2.3), Rallfun-v40, tidymodels (1.0.0), tidyverse (1.3.1), vip (0.3.2) and included training, tuning, and testing as well as visually representing the results.

## Results

### Machine Learning

Although only *R*^2^ can be used to assess a model’s performance on different data sets (e.g., faking high scores vs. faking low scores; see, e.g., Chicco et al., 2021), we nevertheless present all the common performance indices in our results to demonstrate their convergence (e.g., Ayitey Junior et al., 2023; Iskandaryan et al., 2020; see Table 4).

**Table 4.** Performance Evaluation of the Machine Learning Process.

Algorithms		Performance evaluation					
Personality variable	Faking direction	*N_train*	*N_test*	*R^2^*	*RMSE*	*MAE*	*MSE*
*Elastic net regression*							
IM	Low	70	18	−.30	7.52	5.93	56.48
IM	High	68	18	−.08	9.93	8.43	98.67
SM	Low	70	18	.32	2.94	2.39	8.62
SM	High	68	18	.11	3.40	2.85	11.54
*Random forest regression*							
IM	Low	70	18	−.09	6.87	5.19	47.18
IM	High	68	18	.07	9.22	8.18	85.03
SM	Low	70	18	.10	3.38	2.67	11.41
SM	High	68	18	.04	3.53	2.99	12.46

*Note.* IM = impression management; SM = self-monitoring; *N_train* = number of participants in the training data set; *N_test* = number of participants in the testing data set; *R*^2^ = relative improvement in prediction over the mean model; *RMSE* = root mean square error; *MAE* = mean average error; *MSE* = mean square error.

Here, we explain the meaning of the performance indices by giving an example. Using SM and the faking of low scores in elastic net regression as an example, *R*^2^ was .32, *RMSE* was 2.94, *MAE* was 2.39, and *MSE* was 8.62. Thus, when elastic net regression was used and low scores were faked, 32% of the variation in SM could be predicted by the response pattern (68% could not be predicted by the response pattern). According to Cohen (1988), *R*^2^ = .26 represents a large amount of explained variance. *RMSE*, *MAE*, and *MSE* also indicated only a small difference between the prediction results and the true values and thus indicated a good model fit. For comparison, *RMSE*, *MAE*, and *MSE* could have increased in this particular model to *RMSE* = 18, *MAE* = 18, and *MSE* = 324. Thus, the *RMSE* did not exceed 16% of the *RMSE* that would have been possible, *MAE* did not exceed 13% of the maximum *MAE*, and *MSE* did not exceed 3% of the maximum *MSE*.

#### Can Individuals’ IM Scores Be Revealed by Differences in Faking Behavior?

##### Elastic Net Regression

*R*^2^ was −.30 when *low scores* were faked and −.08 when *high scores* were faked (see Table 4). A negative *R*^2^ value indicates a poor model fit (Kvålseth, 1985). *RMSE* was 7.52 when *low scores* were faked and 9.93 when *high scores* were faked. *MAE* was 5.93 when *low scores* were faked and 8.43 when *high scores* were faked. *MSE* was 56.48 when *low scores* were faked and 98.67 when *high scores* were faked.

Thus, the performance evaluation indices from elastic net regression showed that individuals’ IM could not be revealed from differences in their response patterns, as their response patterns were quite similar. The similarity of their response patterns held true for both faking directions (i.e., irrespective of whether high or low scores were supposed to be faked).

##### Random Forest Regression

*R*^2^ was −.09 when *low scores* were faked and .07 when *high scores* were faked (see Table 4). Again, the negative *R*^2^ reveals a poor model fit (Kvålseth, 1985). The *R*^2^ of .07 is considered a small amount of explained variance (Cohen, 1988). *RMSE* was 6.87 when *low scores* were faked and 9.22 when *high scores* were faked. *MAE* was 5.19 when *low scores* were faked and 8.18 when *high scores* were faked. *MSE* was 47.18 when *low scores* were faked and 85.03 when *high scores* were faked. Thus, the performance evaluation indices from random forest regression showed that, in general, individuals’ IM could not be revealed by differences in their response patterns when faking on the E scale. Their response patterns were quite similar for the faking of low scores. However, they were somewhat different for the faking of high scores. Thus, on the basis of individuals’ response patterns, the random forest regression revealed individuals’ IM to a small extent when high extraversion scores were faked.

Taken together, the performance evaluation indices of both algorithms largely converged. They showed that, in most cases, individuals’ IM could not be revealed by differences in their response patterns when faking on the E scale. Their response patterns were quite similar for both faking directions (i.e., irrespective of whether high or low scores were supposed to be faked), except when random forest was used in faking high conditions. Thus, the response patterns of individuals with higher IM scores and those with lower IM scores did not differ much, neither when *faking of high nor when faking low scores* on the E scale.

#### Can Individuals’ SM Scores Be Revealed by Differences in Faking Behavior

##### Elastic Net Regression

*R*^2^ was .32 when *low scores* were faked and .11 when *high scores* were faked (see Table 4). Thus, *R*^2^ was related to a high or moderate amount of explained variance (Cohen, 1988). *RMSE* was 2.94 when *low scores* were faked and 3.40 when *high scores* were faked. *MAE* was 2.39 when *low scores* were faked and 2.85 when *high scores* were faked. *MSE* was 8.62 when *low scores* were faked and 11.54 when *high scores* were faked. Thus, the performance evaluation indices from elastic net regression showed that individuals’ SM could be revealed by the differences in their response patterns. This was true for both faking directions (i.e., irrespective of whether high or low scores were supposed to be faked).

##### Random Forest Regression

*R*^2^ was .10 when *low scores* were faked and .04 when *high scores* were faked (see Table 4). Thus, *R*^2^ was related to a small amount of explained variance (Cohen, 1988). *RMSE* was 3.38 when *low scores* were faked and 3.53 when *high scores* were faked. *MAE* was 2.67 when *low scores* were faked and 2.99 when *high scores* were faked. *MSE* was 11.41 when *low scores* were faked and 12.46 when *high scores* were faked. The performance evaluation indices from random forest regression thus mirrored the results from elastic net regression.

Taken together and as expected, the performance evaluation indices for both algorithms showed that individuals’ SM could be revealed by the differences in their response patterns when faking low scores and when faking high scores. Thus, for SM, individuals’ response patterns differed when we investigated the faking of high and low scores on extraversion. Elastic net regression clearly outperformed random forest regression.

### Feature Importance Analyses

So far, the results have demonstrated that there are differences in response patterns under faking that are related to individuals’ SM but were largely unrelated to individuals’ IM. The exception (i.e., the small relationship between response patterns under the faking of high scores and individuals’ IM when using random forest regression; *R*^2^ = .07) might be explained by the fact that faking direction matters (e.g., Bensch et al., 2019). Nevertheless, in each condition, elastic net regression clearly outperformed random forest regression. Thus, in following Owens et al. (2022), we chose to focus on feature importance from the elastic net regression because it yields the most interpretable coefficients. We also chose to do so because, in contrast to elastic net regression, random forest regression has been described as representing black box methods (e.g., Vijayakumar & Cheung, 2018).

#### How Do Response Patterns Differ by Individuals’ SM?

**Figure 4.**
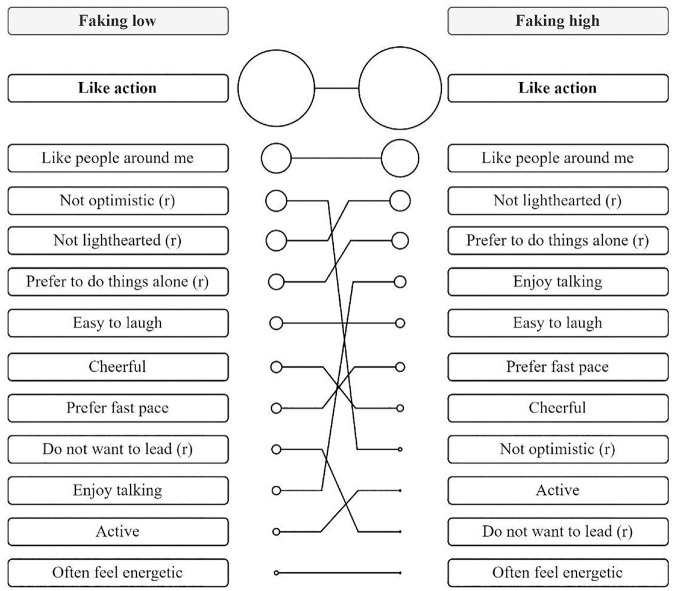
Changes in the Rank Order of Feature Importance With Respect to Faking Direction *Note*. The importance of features (i.e., items) is represented with respect to faking direction (i.e., faking low scores vs. faking high scores) in a descending order. The size of the point and the order indicate the importance of the respective features (i.e., items). The most important feature is set in bold.

To gain better insights into the differences in response patterns, we used feature importance analyses from elastic net regression and investigated *how* individuals’ response patterns differed under faking according to their SM scores.^15^ The feature importance analyses concerning the faking of low scores versus the faking of high scores on the E scale are plotted in Figure 3. The first result is that there was one item that really stood out because it was most important for revealing individuals’ SM. However, Figure 3 also shows that there is an important difference with respect to faking direction. In most cases, the importance of the remaining items differed with respect to faking direction. Thus, a second result is that whether or not items are considered relevant for faking depends on the faking direction. Figure 4 provides additional insights by showing that the rank ordering of the features differed between faking directions. For example, the feature “not optimistic,” which was the third most important feature when low scores were faked, was in ninth place in the importance ranking when high scores were faked. Thus, a third result on how the response patterns differed between individuals with high scores on SM from those with low scores on SM was the following: Some responses were considered to be more or less important to fake by individuals regardless of faking direction. Others, however, were considered relevant for one faking direction but less relevant for the other faking direction (which led to rank-order changes; see Figure 4). Thus, we present the results hereafter with respect to faking direction.

##### The Most Important Features When Faking Low Scores

Concerning SM, the feature importance analyses showed that the most important feature for revealing individuals’ SM was the item that asked whether the participant likes action when individuals were asked to fake low scores. In line with this finding, the unstandardized regression weights showed that the strongest predictor of individuals’ SM when faking low scores was the item “like action” (see Table 5 concerning the *B*s on all items). When the difference score on this item increased by 1, the SM score increased by 1.39. The remaining items had comparably small impacts. For example, when the difference score on the second most important item “like people around me” increased by 1, the SM score increased by 0.54, and when the difference score on the least important item “often feel energetic” increased by 1, the SM score increased by 0.07 (see Table 5). In other words, individuals with higher scores on SM most prominently faked more strongly on the item “like action” than those with lower scores on SM when they were asked to fake low scores on the E scale. For the remaining items, there were comparably smaller differences with respect to faking.

**Table 5.** Unstandardized Regression Weights for the Features With Respect to Faking Direct.

Faking direction	Features	*b*
Faking low	**Like action**	**1.39**
	Like people around me	0.54
	Not optimistic (r)	−0.38
	Not lighthearted (r)	0.37
	Prefer to do things alone (r)	−0.28
	Easy to laugh	−0.23
	Cheerful	0.20
	Prefer fast pace	0.18
	Do not want to lead (r)	0.16
	Enjoy talking	0.15
	Active	−0.12
	Often feel energetic	0.07
Faking high	**Like action**	−**1.50**
	Like people around me	−0.68
	Not lighthearted (r)	−0.37
	Prefer to do things alone (r)	0.30
	Enjoy talking	0.21
	Easy to laugh	−0.16
	Prefer fast pace	−0.16
	Cheerful	−0.12
	Not optimistic (r)	−0.06
	Active	0.01
	Do not want to lead (r)	−0.01
	Often feel energetic	0.00

*Note.* Features are arranged in order of descending importance. Features in bold indicate the most important features for predicting fakers’ scores on SM.

##### The Most Important Features When Faking High Scores

As was the case when individuals were asked to fake low scores, the feature importance analyses also showed that the most important feature for revealing individuals’ SM was the item that asked whether the participant likes action when individuals were asked to fake high scores. In line with this finding, the unstandardized regression weights showed that the strongest predictor of individuals’ SM when faking high scores was the item “like action” (see Table 5 concerning the *B*s on all items). When the difference score on this item increased by 1, the SM score decreased by −1.50. The remaining items had comparably small impacts. For example, when the difference score on the second most important item “like people around me” increased by 1, the SM score decreased by −0.68, and when the difference score on the least important item “often feel energetic” increased by 1, the SM score increased by 0.00 (see Table 5). In other words, individuals with higher scores on SM most prominently faked less strongly on the item “like action” than those with lower scores on SM when they were asked to fake high scores. For the remaining items, there were comparably smaller differences with respect to faking.

## Discussion

Can fakers’ personalities (i.e., IM or SM) be revealed by the traces that they leave when faking, just like perpetrators’ traces in forensic psychology help reveal the perpetrators? Research has revealed contradictory results concerning IM and SM’s relationships to faking. The contradictory results might be explained in part because different studies have given individuals different tests to fake and different faking directions (to fake low scores vs. high scores). Importantly, whereas past research has focused on faking by examining *test scores*, recent advances have suggested that the faking process could be better understood by analyzing individuals’*responses on an item level*. Thus, we followed recent research and focused on the item level to examine whether fakers leave different traces (i.e., different response patterns when faking on an E scale) that can be used to predict their IM or SM. We compared two machine learning algorithms (elastic net regression and random forest regression). We also compared the results with respect to faking direction. We used a well-established self-report measure as the to-be-faked test. Last but not least, we advanced the understanding of faking and its detection by using a bottom-up approach. This approach builds on machine learning to detect which of a scale’s *items* are more likely to be faked and in what direction (Calanna et al., 2020; Röhner et al., 2013, 2022). We examined the items where the most faking occurred (i.e., a data-driven approach) and tested whether we could use the responses on these items to distinguish higher from lower IM or SM scorers to obtain additional insights into traces of faking.

Tying our study back to the scenario of the forensic psychologist: Are we or are we not able to reveal perpetrators’ (fakers’) personalities on the basis of their traces from a crime scene (response patterns under faking)? Concerning IM, our results showed that individuals in general had similar response patterns when they faked, irrespective of their IM scores.^16^ For SM, elastic net and random forest regression converged in revealing that individuals higher on SM differed from individuals lower on SM in how they faked. Feature importance analyses showed that whereas some items were faked differently by individuals with higher versus lower scores on SM, others were faked similarly. Our results imply that analyses of response patterns offer valuable new insights into the faking process.

### Individuals With Higher Versus Lower IM Show Comparable Faking Behavior on the Level of Response Patterns

IM scales are purposely aimed at detecting individuals who are prone to deception. Individuals with higher scores are considered to habitually present an overly positive image of themselves (Paulhus, 2017). Given their declared goal, individuals with higher scores should have differed from individuals with lower scores in their faking in our study. However, the present results showed that the differences were negligible, implying that individuals with higher and lower scores on IM behaved similarly under faking instructions (regardless of whether they tried to fake high or low scores on extraversion). Put simply, the scale failed to detect specific faking patterns that could reliably distinguish individuals with higher IM scores from those with lower IM scores. Although the failure of the scale does not agree with its intended purpose as a measure of faking tendencies, this finding is consistent with recent developments that were based on other research approaches, all converging to suggest that IM scales are ineffective as lie scales (Connelly & Chang, 2016; de Vries et al., 2014; Uziel, 2010, 2014). Whereas these previous studies focused on analyses of a focal scale’s *test score*, the present study adds new evidence that was focused on the *item level*, thereby addressing this question from a different level of analysis.

### Individuals With Higher Versus Lower SM Show Different Faking Behavior on the Level of Response Patterns

The picture was different for SM. Individuals with higher SM scores are considered social chameleons, able to modify and adjust their responses to their social environment (Day & Schleicher, 2006; Snyder, 1974). Past research has been inconsistent in associating SM with faking, and the present analyses shed light on it from the perspective of item-based analyses. Results showed that when this analytic approach is adopted, differences emerge, and individuals who score higher on SM can be differentiated from those who score lower on the basis of their faking.

The feature analyses revealed that the responses of individuals with higher scores on SM were especially likely to differ from those with lower scores on SM on one item (i.e., “like action”) when faking low scores and when faking high scores. However, there were differences with respect to faking direction in the importance of most of the remaining items. This finding is in line with previous research that has demonstrated that the faking of high scores and the faking of low scores are two distinct processes (e.g., Bensch et al., 2019; Röhner et al., 2022).

The analyses also revealed rank-order changes between the to-be-faked items with respect to the faking direction (i.e., faking low vs. faking high scores). Thus, albeit some responses were considered to be more or less important to be faked by individuals regardless of the faking direction (e.g., “like action” was the most important item for both directions; “often feel energetic” was not important for either faking direction), others were considered to be relevant for one faking direction but less relevant for the other. An example of such a difference in importance is the item “not optimistic,” which was the third most important feature when low scores were faked but was in ninth place in the importance ranking when high scores were faked.

For the faking of low and high scores, the most sensitive item on the E scale was “like action.” Individuals with higher scores on SM exhibited stronger faking on this item than individuals with lower scores on SM when asked to fake low on extraversion, whereas the opposite was true when they were asked to fake high on extraversion. We believe that the centrality of this item in the definition of SM is what made it a salient candidate for faking. Individuals with higher and lower scores on SM both had a particularly strong focus on “like action” and addressed it while faking their responses. In addition, not only personality but also faking direction plays a role in decisions about which items are relevant for faking. This finding could inform and direct future explorations of faking by providing information about which items are likely to be at the core of faking in the context of a given trait.

### Theoretical and Practical Implications

The results of the current study have several implications. First, the results add to previous findings by demonstrating that, even at the item level, IM is not associated with faking behavior, and thus, the results provide further evidence in support of the recommendation that IM scales should not be used to “control” for *faking*, even though this procedure has been used in applied settings a lot (Goffin & Christiansen, 2003). Contributing to the theoretical framework of faking, this finding supports earlier research in demonstrating that the idea of bias-prone individuals, which has already attracted considerable criticism, may indeed be inappropriate, at least under circumstances where all individuals could be equally motivated to fake. Practically, this finding underpins the idea that the approach of using “lie scales” is largely ineffective and that these scales are not effective at differentiating faking from nonfaking (e.g., Uziel, 2010, 2014). Thus, the item-level-based analyses and data-driven machine learning approach may serve to further validate measures of faking. This point is especially important given the fact that, in the current literature on personnel selection, the approach to use “lie scales” is still suggested.

Second, the results also show that SM impacts faking on the item level and that there are only a few items that are relevant for differentiating between fakers with higher SM and those with lower SM. This finding provides further support for recent research (e.g., Brown & Böckenholt, 2022) that demonstrated that faking typically takes place *on the item level* (i.e., individuals fake with respect to item content and not just generally on all the items on a scale), although, of course, there may also be individuals who fake in a more blatant way by choosing only extreme responses on all items (Levashina et al., 2014). Nevertheless, the present investigation adds to previous knowledge by providing insights into the “sensitive spots” of a given questionnaire and therefore advances the theoretical framework of faking. Individuals with higher scores and lower scores on SM do not simply fake more or less strongly on all items but select some items and answer them differently while faking. Thus, basic research on faker profiles should consider this point. Our analyses offer a more fine-grained test of faking, which could also be more effective at detecting the weak points of existing surveys. The findings are also relevant for applied settings (e.g., selection procedures). Given that selection procedures often rely on a given set of scales, detecting expected faking patterns and associating them with validated individual-level constructs of faking propensity could be very helpful in improving the reliability of faking detection (and consequently of selection processes). For example, if people differ in their faked response patterns on the basis of their personality characteristics, future faking indices should probably consider this point. Actually, faking detection usually does not work without reservations (Röhner et al., 2022). One reason for misclassification might be that response pattern differences that are based on personality have not been considered so far. The need to develop valid faking detection for applied settings has been substantiated by research that has documented the value of personality questionnaires in diverse contexts (e.g., work performance: Barrick & Mount, 1991; health: Lahey, 2009). Unfortunately, their utility under certain condition (e.g., Ziegler et al., 2012) may be compromised by the ease with which they can be faked. Thus, the ability to handle faking is a central hurdle that must be jumped (Ziegler et al., 2012). Note that, the risk of faking is not only related to high-stakes settings (e.g., personnel selection; Morgeson et al., 2007). Even when individuals are not intrinsically inclined to bias their self-reports, they are likely to change their reports if they assume that others also give biased answers (Grover, 1993). Taking personality differences into account when developing faking indices might help improve faking detection.

Third, the results show that faking direction plays a role with respect to which items are especially important (i.e., are prone to faking). For example, the feature “not optimistic,” which was the third most important feature when low scores were faked, was in ninth place in the importance ranking when high scores were faked. Thus, whereas some items were more important for faking regardless of faking direction, others were considered to be relevant for one faking direction but less relevant for the other faking direction. Not only might these findings explain some of the contradictory results from previous studies that focused primarily on one faking direction (either high or low), but they are also in line with the suggestion that the faking of high and low scores might be distinct constructs (e.g., Bensch et al., 2019; Röhner et al., 2022). Considering applied settings, this result indicates that faking manifests in different items with respect to faking direction, a finding that implies that, to detect faking, it is possible that separate faking indicators are necessary for fakers of low scores and fakers of high scores (an approach that is already used in behavioral measures; e.g., Röhner et al., 2023).

### Limitations and Further Directions

Our study has potential limitations regarding the generalizability of the results. First, we examined faking on one measure only (i.e., an E scale). Some research has indicated that faking varies with respect to the to-be-faked construct at least under some circumstances (e.g., Röhner et al., 2022). Future research should extend our findings to other measures.

Second, we restricted ourselves to two personality variables that have been most frequently suggested to impact faking in faking models. However, there are other variables that could be investigated in this context (e.g., self-deception, Machiavellianism, narcissism, or psychopathy).

Third, our study focused on SM as a unitary construct (Fuglestad & Snyder, 2009). Although there are alternative conceptualizations of SM, we believe that our approach is adequate in the present context.^17^ Notwithstanding, a recent body of work has pointed to an alternative bivariate model of SM compromised of two orthogonal factors (i.e., acquisitive SM and protective SM; see Wilmot, 2015; Wilmot et al., 2016), which show different correlations with related scales (e.g., Machiavellianism: Rauthmann, 2011; authenticity: e.g., Laux & Renner, 2002; Renner et al., 2004). Nevertheless, research has also indicated parallel findings for SM in the univariate model and bivariate model (e.g., Leone, 2022). Yet, as Fuglestad et al. (2020) stated, “much theoretical and empirical work remains to be done to fully understand both protective and acquisitive self-monitoring” (p. 231).

Future research should consider this alternative bifactor model more while studying the role of SM in faking behavior. For example, it is plausible that these factors have different relationships with faking motives (i.e., whereas acquisitive SM may be related to faking in the sense of obtaining rewards [e.g., social status], protective SM may be more strongly related to avoiding costs [e.g., social rejection]). This topic goes beyond the scope of our research but is an important avenue of future research.

Fourth, with elastic net and random forest, we analyzed the data with two well-established algorithms that have also been successfully applied to faked data (e.g., Calanna et al., 2020; Röhner et al., 2022). However, future research might add other algorithms. For example, neuronal networks, which try to simulate the structure of the human brain, could be considered, especially when other data dimensions are included (e.g., other to-be-faked constructs), thus increasing the complexity of the analyses. However, such analyses usually need large data sets, and the results are mostly black boxes (e.g., Adadi & Berrada, 2018).

Fifth, we followed a new data-driven approach to investigate the association between personality variables and faking. Although there is theoretical justification for our approach, future studies are needed to proof for the generalizability of the results.

Last but not least, our manipulation was based on instructing students to fake (vs. not). One might argue that instructed faking may induce some kind of artificial faking. Of course, this possibility cannot be ruled out. However, it is important to note that researchers do not have valid faking detectors at the moment (e.g., Röhner et al., 2022), and thus, there is no way to clearly differentiate between fakers and nonfakers in applied settings, making this manipulation a necessary precondition for this type of research. Currently, data sets with instructed faking best fulfill the properties that are required to investigate faking with machine learning. Nevertheless, whether the results are generalizable to samples from other situations in which faking occurs naturally (or to other populations) is a question for future research.

## Conclusion

The present investigation showed that machine learning can be applied in the service of investigating response patterns for faking at the item level. It uncovered some of the characteristic response patterns that individuals with higher (vs. lower) scores in central traits adopt when faking their reports. Although the findings do not imply that individuals with higher scores on IM or SM are fakers, they imply that fakers with higher or lower scores on SM leave different traces, and thus, the extent of faking on those items is a good marker for some traits (SM) but not others (IM). These findings add insights into the traits involved in faking and potentially improve our ability to further investigate and detect it.

## Notes

1. To overcome the problems that come with this approach, many other approaches have been suggested, each facing its own difficulties. Another approach that is based on the congruence model of faking suggests using response times to detect faking (e.g., Holden et al., 1992). Research has indicated that the inspection of response latencies can detect faking to some extent (e.g., Holden & Lambert, 2015). However, this approach seems to be restricted to certain conditions (Röhner & Holden, 2022). Researchers have also provided faking indices for selected measures that can be used to detect faking (e.g., Schmidt-Atzert et al., 2004), but by nature, they are restricted to those measures.

2. Such conditions are usually met in faking research (e.g., high treatment effects between faking and nonfaking conditions). This issue is thoroughly explained in the Method section.

3. One might wonder whether previous research justifies the approach to predict IM or SM from response patterns. Please note that from a theoretical stance, our approach is not unusual. For example, it is justified by the fact that a common way to validate IM scales is by asking people to fake, and then measure their levels of IM (i.e., scores on the IM scales). This approach (where IM is a dependent variable) is often applied to prove that IM is a valid measure of faking (i.e., a good independent variable; e.g., Paulhus et al., 1995; Stöber, 2001). We do the same thing, but employed a more sophisticated approach that targets the items where faking was done (as opposed to the mean difference).

4. Response pattern is defined as a participant’s entire set of item responses (Calanna et al., 2020).

5. In principle, either test scores or individuals’ responses to items can be used to compute these differences. Considering the argumentation above, we used the responses to each item here.

6. One can think of this approach as similar to the work of a forensic psychologist who tries to create a profile of a perpetrator by predicting it from traces at the crime scene. The forensic psychologist tries to draw conclusions about the personality of the perpetrator on the basis of the traces that the perpetrator has left. Similarly, we tried to reveal whether faking behavior can be used to draw conclusions about the personalities of fakers.

7. Instructing individuals to fake represents the most common methodology for investigating faking behavior (Smith & McDaniel, 2012), as this methodology provides valuable insight into the extent to which people can fake and into the strategies people apply when asked to fake (see Smith & McDaniel, 2012).

8. Due to space restrictions in the reference list, we add these references in our Supplement on the OSF (https://osf.io/ujvwd/).

9. The ancmg1.power function is stored on the OSF (https://osf.io/ujvwd/).

10. We analyzed whether their level of faking motivation (ranging from 1 = *very low* to 5 = *very high*) was different with respect to faking condition (low vs. high). The level of motivation was comparable between the faking low and faking high conditions (*M*_faking low_ = 4.16 and *M*_faking high_ = 4.31), *t*(169) = −1.32, *p* > .05.

11. We decided to base our means on summation scores and not on average scores to follow the procedures used by the test authors and in previous literature for reasons of comparison. Nevertheless, it is easier to understand the distribution on a scale when the means are based on average scores. Thus, for each scale, we added *M* and *SD* based on average scores (i.e., *M*_a_ and *SD*_a_) and identified *M* and *SD* based on summation scores as *M*_sum_ and *SD*_sum._

12. We also computed the difference scores on a test score level for additional analyses that included the test score (see Figure S1 and Table S2 in the Supplement).

13. We analyzed the reliability at a test score level as a rough proxy. Given that the preliminary analysis revealed unequal variances, we used Trafimow’s (2015) Equation 9. The results indicated that the difference score was reliable (i.e., *r*_diff_ = .95).

14. We had also previously used a classification approach in machine learning (algorithm: logistic regression). The results were in line with those from the regression approaches presented here and can be obtained from the lead author upon request.

15. Although we followed recommendations from recent research to investigate faking at the item level (Brown & Böckenholt, 2022), we also included the difference score of the test scores as an additional feature in additional analyses. As expected, the test score was never the most important feature, and thus, it was outperformed by the relevance of single items (see Figure S1 and Table S2 in the Supplement on the OSF for these additional analyses).

16. An exception involved the faking of high scores when random forest regression was used.

17. The author of the SM scale we used in our study recommended that SM be assessed as a unidimensional construct (i.e., univariate model of SM; Graf, 2004) for several reasons: (a) it was constructed this way (see Graf, 2004); (b) although the scale allows three factors to be assessed, it measures only one dimension (Gangestad & Snyder, 2000); and (c) using all the items in one scale allows for better prediction of criteria than using subscales (Snyder, 1987). Moreover, the univariate model of SM has been the dominant interpretative paradigm in the literature for over 4 decades, whereas research that has emphasized more than one factor has often not converged into an alternative factor structure that was largely accepted by all scholars (e.g., two factors: Sparacino et al., 1983; three factors: Briggs et al., 1980; or even four factors: Gabrenya & Arkin, 1980). Thus, in a long-standing conceptual and psychometric debate, researchers have advocated analyzing SM from multiple perspectives (e.g., Gangestad & Snyder, 2000; Wilmot et al., 2016).
